# The Phospholipase D2 Knock Out Mouse Has Ectopic Purkinje Cells and Suffers from Early Adult-Onset Anosmia

**DOI:** 10.1371/journal.pone.0162814

**Published:** 2016-09-22

**Authors:** Matthieu M. Vermeren, Qifeng Zhang, Elizabeth Smethurst, Anne Segonds-Pichon, Heinrich Schrewe, Michael J. O. Wakelam

**Affiliations:** 1 Inositide lab, the Babraham Institute, Babraham, Cambridge, CB22 3AT, United Kingdom; 2 Department of Developmental Genetics, Max-Planck Institute for Molecular Genetics, 14195, Berlin, Germany; The University of Queensland, AUSTRALIA

## Abstract

Phospholipase D2 (PLD2) is an enzyme that produces phosphatidic acid (PA), a lipid messenger molecule involved in a number of cellular events including, through its membrane curvature properties, endocytosis. The PLD2 knock out (PLD2KO) mouse has been previously reported to be protected from insult in a model of Alzheimer's disease. We have further analysed a PLD2KO mouse using mass spectrophotometry of its lipids and found significant differences in PA species throughout its brain. We have examined the expression pattern of PLD2 which allowed us to define which region of the brain to analyse for defect, notably PLD2 was not detected in glial-rich regions. The expression pattern lead us to specifically examine the mitral cells of olfactory bulbs, the Cornus Amonis (CA) regions of the hippocampus and the Purkinje cells of the cerebellum. We find that the change to longer PA species correlates with subtle architectural defect in the cerebellum, exemplified by ectopic Purkinje cells and an adult-onset deficit of olfaction. These observations draw parallels to defects in the reelin heterozygote as well as the effect of high fat diet on olfaction.

## Introduction

Phospholipase D (PLD) is a signalling enzyme that catalyses the hydrolysis of the membrane lipid phosphatidylcholine (PC) to generate a soluble choline head group and the lipid phosphatidic acid (PA)[1–3]. With its small negatively-charged head group and two large acyl chains that confer a “cone-like” morphology, PA triggers a conformational change in the membrane that results in local negative folding. PA is also a bioactive lipid that regulates protein position by attracting their positively charged PA-binding domains to the membrane, and sometimes modulates their activity [4–6]. The PLD gene family comprises six members, however only PLD1 and PLD2 have been clearly demonstrated to hydrolyse PC. Whilst PLD1 is associated with the endoplasmic reticulum, Golgi compartments, secretory granules and lysosomes [7–9], PLD2 has been demonstrated to localise to the plasma membrane [10]. PLD1 and PLD2 both selectively hydrolyse unsaturated or monounsaturated PC species, while other PA producing enzymes such as the diacylglycerol (DAG) kinase isoforms which can use polyunsaturated or saturated substrates, suggesting an exquisite specificity in activity control [11]. Through the use of inhibitors, PLD-derived PA has been reported to be involved in a variety of processes such as receptor endocytosis, vesicle trafficking, cell migration and differentiation (see [3]). Recently, mouse knock outs for PLD1 and PLD2 have been described [12–18]. Loss of allele in the PLD1KO mice attenuates autophagy [15] and alpha IIb beta3 integrin activation is impaired, which is suggested to lead to a high shear thrombus defect [16] and protection against lung tumour metastasis [14]. PLD2KO shows attenuation of Alzheimer Disease pathogenesis by increased resistance to Aβeta oligomer insult and memory deficit rescue [18].

In view of the considerable body of work on PLD2 function [3], we have further analysed a mouse knock out for PLD2 presented earlier in our lab [17]. We have defined PLD2 expression pattern in the normal adult mouse brain and have analysed regions of high PLD2 expression using histology, behaviour and lipidomic techniques. We present evidence that PLD2KO mice have an abnormal PA profile in the brain concomitant with ectopic Purkinje cells and olfactory defects.

## Materials and Methods

### Ethics Statement

All mouse work rigorously followed the British Animal Scientific Procedures Act (ASPA) under licence of the Home Office (UK). Mice were sacrificed following schedule 1 method: CO2 or cervical dislocation.

### LNA In situ hybridisation

PLD2 LNA, /DigN/GGTCTGGGATAAAGGAAAGTTGA/Dig/, scrambled LNA, /Dig/GTGTAACACGTCTATACGCCCA/Dig/, and positive control mmu-mir-124 LNA, /Dig/GGCATTCATTCACCGCGTGCCTTA/Dig, were custom made by Exiqon (Vedbæk, Denmark). In situ hybridisation method was as described in [19]. Sections were counterstained with Hoechst and mounted with the anti-fading agent 2.5% 1,4-Diazabicyclo[2.2.2]octane (DABCO, Sigma)/ 50mM Tris-Cl pH 8.0/ 90% Glycerol pH8.0. They were recorded using a Zeiss Imager.D2 microscope (Zeiss, UK). Images were assembled using the photomontage automatic function of Adobe Photoshop and annotated using the same programme. For the identification of the PLD2-positive brain regions, we used the sagittal adult mouse brain reference from the Allen Brain Atlas (http://mouse.brain-map.org/static/atlas). We examined two 15 weeks old adult mice, one male and one female, and did not find difference between genders.

### Mouse strains

PLD2KO mice were bred on the C57BL^Babr^ background as in [17]. They were crossed with PCP2GFP (B6;FVB-Tg(Pcp2-EGFP)2Yuza/J) [20]or THYYFP (B6.Cg-TgN(Thy1-YFP-H)2Jrs mice (YFP-H) [38]. Mice are fed a normal pellet diet and have access to water *ad libitum*.

### PCP2-GFP and Thy-YFP mouse analyses

Brains of aged matched mice of either sex of PCP2 GFP or Thy YFP WT and PLD2KO were isolated and fixed overnight in 4% PFA/PBS. They were then embedded in 20% gelatin/PBS and fixed for 48h in 4% PFA. Blocks were transferred to PBS and kept at 4°C until sectioning. Brains were sectioned on a Leica VT1000S vibratome in 70 micrometre thick sections. Sections were mounted in DABCO/Tris/glycerol and were analysed using an Olympus 1X81 microscope and FV1000S Leica confocal microscope. Ectopic PCP2-GFP positive Purkinje neurons were counted and quantified per brain. There were 3 wild type of either sex and 3 females and 5 male knock out analysed. Statistical analysis was T-test. All animals were adults and ranged between 10 and 15 weeks of age.

### Behaviour

We assembled two cohorts of gender and aged-matched related mice from our PLD2KO mouse line, derived from heterozygotic crossing. These mice underwent the following behavioural experiments:

1 Modified SHIRPA
  - Modified SHIRPA protocol was as described in [21]
2 Open field
  - Mice were recorded for five minutes exploring an arena 50x50 cm. They were timed for how long it took them to enter a central area, which covers 30x30 cm. Their movements were traced using Noldus ethovision XT (Noldus Information Technology, Walgeningen, the Netherlands).
3 Food burrowing test
  - Mice were used to Cadbury’s chocolate buttons (Cadbury's, Bournville, UK) for a week. They were then placed in a cage with fresh bedding under which a piece of chocolate was hidden. Mice were timed until they found the chocolate or 10 minutes had elapsed.

For SHIRPA, Food burrowing test and Open Field test, mouse cohort was made up of 11 female and 12 male PLD2KO and 11 female and 15 male wild type. Mice tested were between the age of 14 and 20 weeks old.

4 Rotarod
  - A cohort of adult 5 male and 5 female PLD2KO and 2 male and 8 female Wild Type was used. Mice age ranged from 18 to 30 weeks. Mice were tested for five minutes on the accelerating rotarod, speed gradually increased from 3.5 rotations per minute (rpm) to 35 rpm on the fourth minute and then stayed constant for the last minute. Mice were scored according to whether they fell off and how long they stayed on the rotating rod.
5 Odour habituation/deshabituation
  - A cohort of 11 male and 8 female PLD2KO and 6 male and 13 female wild type was tested. For three day running, a cotton bud soaked in water perfumed with orange food flavouring (1:10000) was anchored hanging in the cage of mice being tested, 1 cm above bedding. On the fourth day, the cotton bud was soaked with water perfumed with vanilla (1:10000). One by one, each mouse was recorded for how many times its nose came into contact with the cotton bud. There was no difference between cohorts that had or had not tasted chocolate before.

For the purpose of the habituation-deshabituation experiment and food burrowing tests, a second cohort of younger mice was tested at 11 weeks of age. There were 3 male and 3 female PLD2KO mice and 2 male and 3 female wild type mice.

### c-fos staining

18 weeks old male PLD2KO mice (n = 4) and Wild Type (n = 7) controls were presented with a cotton bud imbibed with vanilla or chocolate. They were left for 30 minutes before being sacrificed and their brain dissected and fixed in 4% PFA/PBS. The brains were then cryosectioned coronally and serial sections immunostained against c-fos (AbCam) and counterstained with DAPI. The mitral cell layer was then analysed and c-fos positive neurons counted.

### Lipidomics

Two cohorts constituting of a total of 12 wild type and 12 knock out adult mice that were age and gender-matched had their brain dissected. Both left and right cortex, hippocampi and olfactory bulbs as well as cerebellum, a region encompassing the caudoputamen, Thalamic and hypothalamic regions and finally a region encompassing the midbrain, pons and medulla were subdissected. Snap-frozen brain samples were pulverised in liquid nitrogen. 0.1mg of each brain tissue sample was extracted and the phosphoinositides derivatised as described by [22]. Phosphoinositides and phosphatidic acid were analysed by mass spectrometry as previously described by [22], making use of 10ng of 16:0/17:0-PA and 10ng of 16:0–17:0-PIP_3_ as internal standards for recovery and quantification of the analysis. Full results are presented in S1 File.

## Results

### Lipidomic profile of brain regions

**Fig 1:**
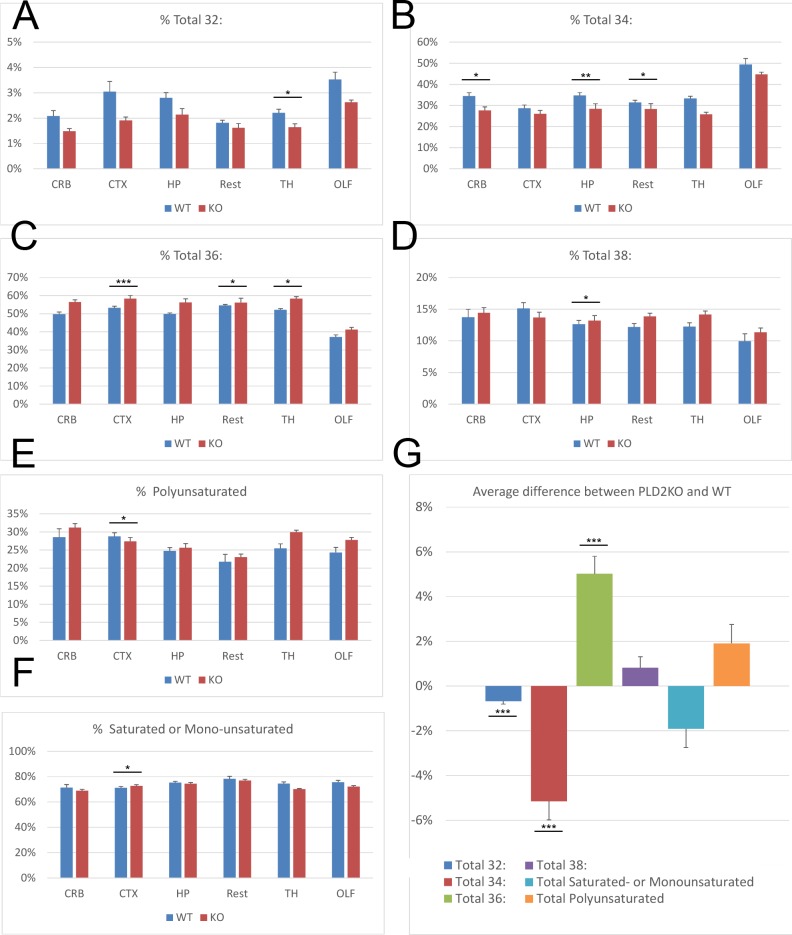
Lipidomic analysis indicates lipid imbalance in PLD2KO mouse brain. **A-F** Lipidomic analysis of six regions of the brain indicate that PLD2KO mice have significant changes in their pools of PA. Percentage of PA species are calculated compared to the total sum of PA species identified. A) 32: species, B) 34: species, C) 36: species, D) 38: species, E) Polyunsaturated species, F) Saturated- and monounsaturated species. *CRB*, Cerebellum; *CTX*, Cortex; *OLF*, Olfactory bulbs; *TH*, Thalamus-Hypothalamus and Striatum; *HP*, Hippocampus; *Rest*, Midbrain-Hindbrain and Medulla. **G** Comparison of difference between percentage of species in WT and PLD2KO indicate that the overall proportion of 32:, 34: and 36: species have significantly changed. A-F significance is calculated by Univariate Analysis of Variance test over two different experiments. G significance is calculated as paired t-test comparing the profile of all brain regions. When p<0.05, *; p<0.01, **; p<0.005, ***

The nervous system is a phospholipid-rich tissue where loss of a lipase is likely to have a significant impact, therefore we focussed initially upon changes in the adult brain. To determine if the loss of PLD2 impacted upon the pool of phosphatidic acid lipids, either quantitatively or qualitatively, lipid mass spectrometry was adopted. The brains of WT and PLD2KO mice were dissected and six different regions: the olfactory bulbs (OLF), cortex (CTX), cerebellum (CRB), hippocampus (HP), a region encompassing the striatum, hypothalamus and thalamus (TH) and finally the combined midbrain, hindbrain and medulla (Rest) isolated. These samples were pulverised under liquid nitrogen, the lipids extracted, separated by HPLC and analysed by mass spectrometry. Full results are found in S1 File. The overall PA content relative to the main lipid content was not depleted in any region, suggesting that the activities of additional PA-producing enzymes, for example PLD1, can compensate for the absence of PLD2-produced PA. It is alternatively possible that PLD2 contribute little to the PA pool in the brain. However, the species profile of the PA pool, which is defined according to the length and saturation levels of the two acyl chains that make the backbone of PA, indicated that there are general and regional differences (see Fig 1). For example, we find significantly less 32: PA in the thalamic region (WT = 2.21%, PLD2KO = 1.63%, P<0.05, Fig 1A). Likewise, we find significant decrease in 34: PA species in the CRB, TH and Rest regions (Fig 1B). If all the results are compared as a whole, we find that PLD2KO mice have a highly significant 5% more of the C36: species in all brain regions (see Fig 1G, p<0.005). Conversely, we find significantly less of the C32: and C34: species (p<0.005). There is therefore a shift from C32: and C34: towards C36:. While C38: species do show an increase, it is not significant. It is worth noting that the overall lipid composition between regions is somewhat homogenous except in the case of the olfactory bulbs. This may be caused by the presence of lipid rich peripheral glial cells in the glomeruli. The differences observed were not specific to saturated or monounsaturated PA species, which are thought to be preferential products of PLD2 (and indeed PLD1) activity [11], since polyunsaturated PAs were overall normal except in CTX and TH (see Fig 1E and 1F). Thus, while there is a quantitatively normal PA pool in PLD2KO brain, its specific acyl chain composition is perturbed by the absence of PLD2, with clear regional differences.

Since the activity of Phosphatidylinositol 4-phosphate (PI4P-5) kinase, the Igamma isoform in particular, can be regulated by changes in PA [23], we considered that a perturbed pool of PA may have an impact on the phosphoinositide lipid species. Consequently, lipids were extracted from the same brain samples as used for PA analysis, derivatised using trimethylsilane and the phosphoinositides identified by mass spectrophotometry [22]. However, we did not detect a difference in phosphatidylinositol 4,5-bisphosphate (PIP_2_) levels between WT and PLD2KO brains.

### PLD2 expression in the brain

**Fig 2:**
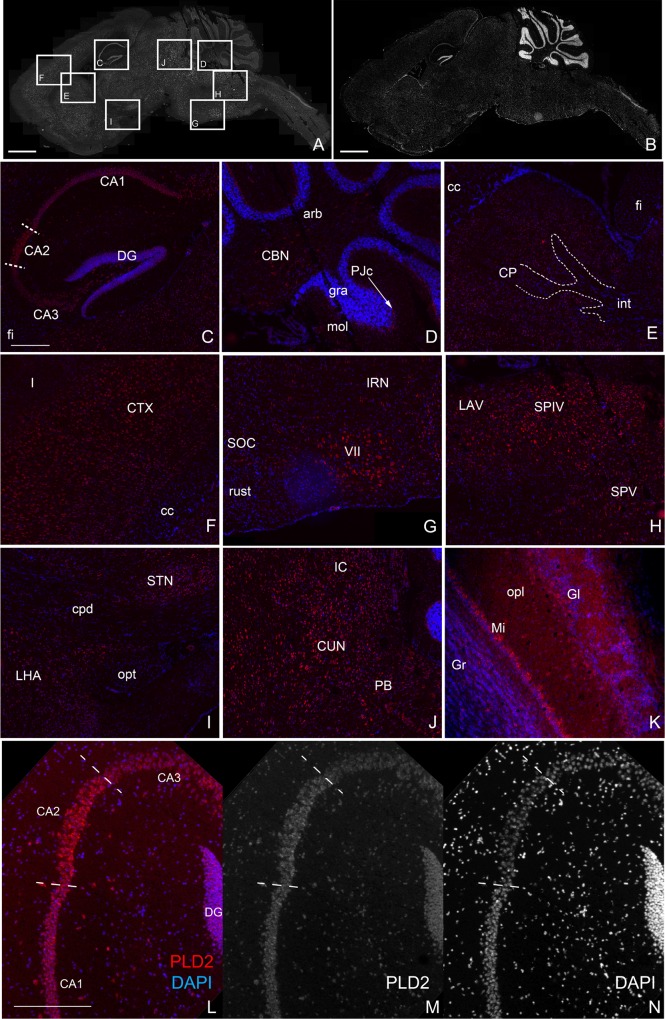
In situ hybridisation reveals widespread expression of PLD2. LNA in situ hybridisation detects PLD2 mRNA in a variety of neuronal but not glial structures in the mouse adult brain. **A, B**: reconstruction of a sagittal section stained with PLD2 LNA (**A**) and counterstained with Hoechst to show overall histology (**B**). Scale bar = 2mm. **C-J**: different regions of the brain magnified from the boxes in (A) with PLD2 LNA (red) and Hoechst counterstain (blue). **C**: Hippocampus; PLD2 is expressed a high levels in the CA pyramidal layers, specially the CA2 field (CA2, defined by dashed lines) and at lower levels in the dentate gyrus (DG) while it’s absent from the fimbria (fi). The lateral dorsal (LD) and lateral posterior (LP) nuclei of the thalamus and the subiculum (sub) also express PLD2. **D**: Cerebellum; high levels of PLD2 are detected in the Purkinje cell layer (PJc, arrow) and the Cerebellar Nuclei (CBN) but not in the glia of the arbor vitae (arb), the molecular layer (mol) and the granular layer (gra). **E**: Caudoputamen (CP), PLD2 is detected at low level in all neurons but not in the surrounding glial-rich fiber tracks of the corpus callosum (cc), fimbria (fi) and internal capsule (int, dashed lines). **F**: Isocortex; layers II-VI of the cortex (CTX) express PLD2 but not the corpus callosum (cc) or the outermost glia-rich layer I (I). **G**: Pons; PLD2 is expressed at high levels in the facial motor nucleus (VII), at lower levels in the superior olivary complex (SOC) and intermediate reticular nucleus (IRN) and not at all in the rubrospinal track (rust). **H**: Medulla; high levels of PLD2 are detected in the spinal vestibular nucleus (SPIV), the spinal nucleus of the trigeminal (SPV) and the lateral vestibular nucleus (LAV) but not in the parvicellular reticular nucleus (PARN). **I**: Hypothalamus; PLD2 positive nuclei of the hypothalamus such as the subthalamic nucleus (STN) and lateral hypothalamic area (LHA) are separated by signal-free glial-rich fiber tracks of the optic nerve (opt) and cerebral peduncle (cpd). **J**: Midbrain; most neurons express PLD2 such as the inferior colliculus (IC) or the cuneiform nucleus (CUN). The parabrachial nucleus also expresses high levels of PLD2. **K**: Olfactory bulb: PLD2 is detected at high levels in the mitral cell layer (Mi) and interspersed in a few interneurons in the outer plexiform layer (opl) but not in the glomerular layer (Gl) or the granular layer (Gr). **L-N**: higher magnification of the CA fields of the hippocampus showing that there is more PLD2 detected in the CA2 field relative to cell content defined by DAPI staining. Scale bars A, B = 2mm C-M = 500 μm

In order to clearly identify which brain region was most likely to be affected by the loss of PLD2, and thus understand if and where the PA imbalance is likely to have an effect, it was necessary to analyse the PLD2 expression pattern. Previously published expression patterns of PLD2, using radioactive in situ hybridisation, antibody staining or Western blotting, have been patchy, lack precision and have been frequently contradictory [10, 24–27]. Data presented in the Allen Brain Atlas are equally inconclusive [28]. We found that traditional in situ hybridisation did not generate reproducible results, possibly because the PLD2 mRNA expression level is low. To circumvent this problem, we adopted a Locked Nucleotide Amplification (LNA)-based approach[19]. This method, combined with tyramide amplification of the signal, is very sensitive and works extremely well with nervous system tissue. The results showed that PLD2 mRNA detection was not ubiquitous and could be observed in diverse regions of the brain (see Fig 2). Remarkably, in contrast to the antibody staining profile presented previously [29], we could not detect any signal in glia-rich regions such as the fimbria, the arbor vitae of the cerebellum, the internal capsule running through the Caudoputamen or the corpus callosum (Fig 2Cfi, 2Darb, 2Eint and 2Fcc). In contrast, most functional regions of the brain expressed PLD2 mRNA to varying degrees, depending on neuron types. For example, the facial motor nucleus expresses more PLD2 than the neighbouring superior olivary complex (Fig 2GVII and 2GSOC, respectively). Likewise, the CA2 region of the hippocampus has noticeably more signal than the surrounding CA1 and CA3 regions, which themselves express far more than the Dentate gyrus where PLD2 is almost undetected (Fig 2C, 2L, 2M and 2N). In three functional regions, the PLD2 LNA signal stood out within anatomically-defined cell types: the pyramidal cells of the CA regions of the hippocampus, the Purkinje cells in the cerebellum and the mitral cells of the olfactory bulbs (Fig 2CCA1, 2CCA2, 2CCA3, 2DPJc and 2KMi). These neurons were clearly surrounded by other non-labelled neurons and glia, highlighting either that PLD2 is not expressed in these cells or at such low level as to be undetectable. Therefore, loss of PLD2 from the hippocampus, cerebellum, and the olfactory bulbs may impact on their function, leading to morphological problems that could be reflected in mouse behaviour.

### Morphological analysis of the PLD2 KO

**Fig 3:**
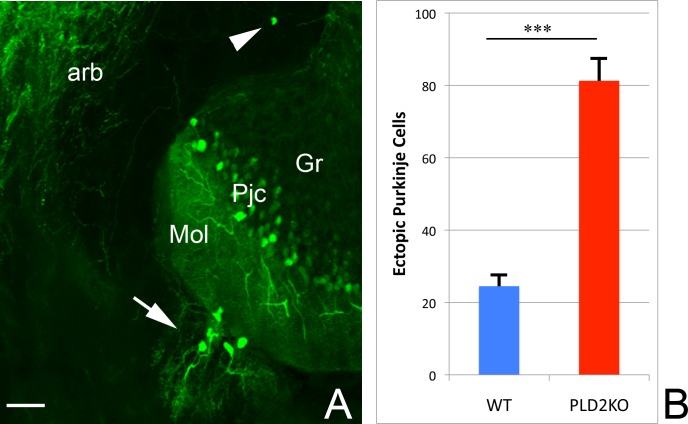
The cerebellar architecture is affected in PLD2KO. A: Normally, the Purkinje cells (Pjc) form a monolayer sandwiched between the granular layer (Gr) and the molecular layer (Mol). In PLD2KO, ectopic Purkinje cells are also found either in the arbor vitae (arb, arrowhead) or clustered on the surface of the molecular layer (arrow). Scale bar = 100μm. B: PLD2KO mice have significantly more ectopic Purkinje cells than WT mice (p<0.005)

We considered if the abnormal PA lipid profile of PLD2KO mouse brain would be reflected in its morphology at tissue and single cell levels. Histological analyses of the PLD2 KO brain using H&E, classical Golgi silver staining or DiI injection methods did not reveal any specific abnormalities: overall architecture of the brain looked normal, the neurons were well distributed in correct positions and their size and density looked normal. However, these methods were limiting since it was not always possible to quickly and reliably follow a single cell from dendritic tip to axon terminal end. To circumvent this problem, we crossed the PLD2KO mice with two strains that express fluorescent markers in neurons that express high levels of PLD2 in the wild type. We used a PCP2-GFP line where GFP expression in the cerebellum is limited to the Purkinje cells and we selected a Thy-YFP line to examine the pyramidal neurons of the hippocampus. While the cerebellum of PLD2KO mouse appeared mostly normal, analysis of the PLD2KO/PCP2-GFP mice revealed a significant and surprising abnormality: we found an increased number of ectopic Purkinje cells in the PLD2KO (WT 24.5 ectopic neurons/cerebellum n = 6 mice, PLD2KO 81.25 ectopic neuron/cerebellum n = 8 mice, p<0.0001, Fig 3). These cells were either embedded within the deep cerebellar nuclei white matter or formed a second molecular layer, including large dendritic arborisation, at the surface of the normal layer. In contrast, we did not observe any significant difference in the hippocampi of the Thy-YFP mice between WT and PLD2KO.

### Behaviour

Biochemical analyses indicated that the brain of PLD2KO mice has an abnormal PA profile. Furthermore, PLD2KO mice have abnormal and mislocated Purkinje cells, suggesting that their function may be affected. Whilst we did not observe an untoward morphology in the mitral cells or the CA pyramidal neurons, it remained possible that they had an abnormal physiology. Mitral cells are necessary for olfaction, CA pyramidal neurons are involved in memory processing and spatial exploration and Purkinje cells are required for fine motor skills. Thus we decided to analyse behaviour in the PLD2KO mice.

**Fig 4:**
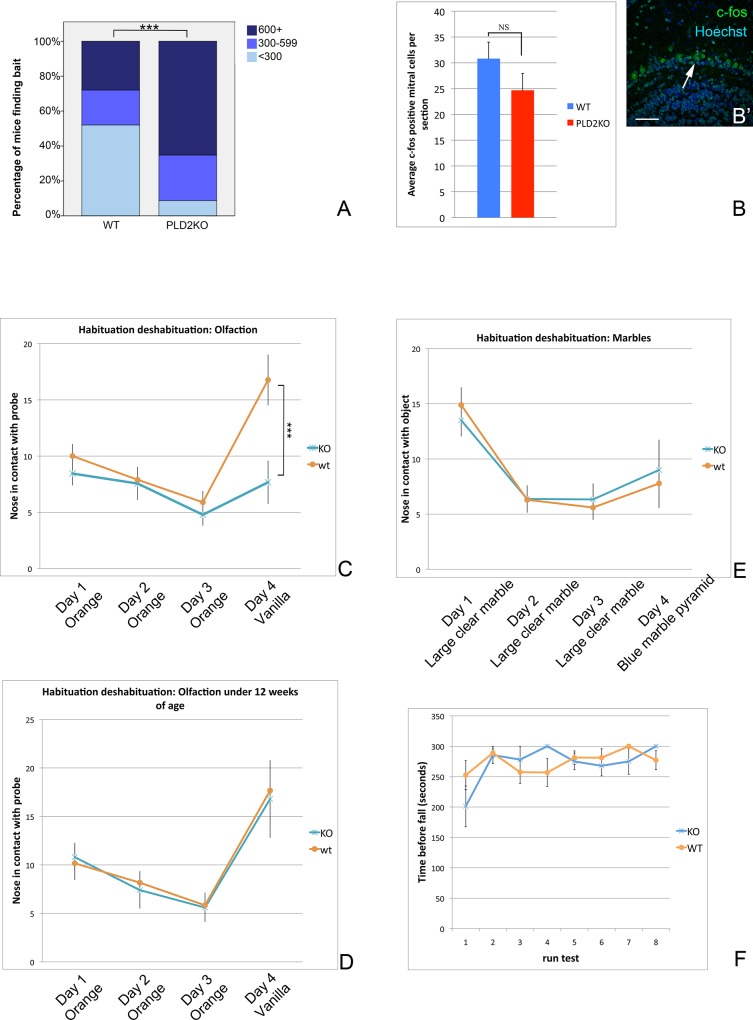
PLD2KO mice suffer from late-onset anosmia. Independent tests indicate that PLD2 KO mice have olfaction defect and that these defects are age-dependent and begin after 13 weeks of age. **A**: Buried food test; mice were timed for the retrieval of a small piece of chocolate hidden under bedding. In 72% of trials WT mice find the bait in less than 600 seconds, in contrast in only 34.8% of trials are PLD2KO mice able to find it; p<0.005. **B and B’**: Immunostaining of olfactory bulbs cryosections against c-fos (green) and counterstained with Hoechst (blue) identifies activated mitral cells in the mitral cell layer (arrow). Scale bar = 50μm. There is no difference in the number of active, c-fos positive, mitral cells between WT and PLD2KO male mice (blue and red, respectively) that have been challenged with vanilla or chocolate 30 minutes prior to sampling. **C**: Habituation-deshabituation; mice were used for three days to a cotton bud smelling of orange and on the fourth day were presented with a cotton bud smelling of vanilla. While PLD2KO mice (blue line) do not differentiate the smell, WT mice (orange line) show a regain interest in the new odour; p<0.001. **D**: The difference in habituation deshabituation response to olfactory cues is age dependent as there is no difference between WT and PLD2KO in mice that are 11 weeks of age, unlike the 14 week-old cohort presented in (C). **E**: When different objects that have the same smell are presented to the mice, WT and PLD2KO react similarly. **F**: PLD2KO mice are as good as WT mice at learning and sustaining the accelerating rotarod test.

Overall, PLD2KO mice live as long as their WT counterparts and do not show any striking behavioural phenotype: they appear healthy, reproduce normally and have no unusual morbidity or weight problems. Indeed, SHIRPA (SmithKline Beecham, Harwell, Imperial College, Royal London Hospital, Phenotype Assessment) index analysis, a set of standardised behavioural observations and tests that measure grooming, activity levels, respiration, gait, muscle tone, reflexes and aggression [21, 30] did not detect any behaviour out of the norm. Nevertheless, SHIRPA comprises a range of observation in an unchallenging environment and if there is a subtle behavioural phenotype this analysis could fail to detect. We tested the mice with the rotarod (Fig 4F) and open field paradigms [31, 32] but again these did not reveal any difference between WT and PLD2KO mice, suggesting that sense of balance and exploratory behaviours are normal. Olfaction was then examined using the buried food test, where the mice are timed to find a small piece of chocolate hidden under clean bedding. Unexpectedly, PLD2KO mice had significant difficulty in finding the bait with 26.1% of them taking more than five minutes and 65.2% not finding it at all (Fig 4A). In contrast, 52% of the wild-type mice found the chocolate within five minutes and a further 20% between five and ten minutes (p<0.005). This was surprising since PLD2KO mice were as fast as the WT at eating non-hidden chocolate. Therefore, either PLD2KO mice suffer from anosmia, the inability to smell, or they are unable to localise the origin of a smell. To differentiate between these two possibilities, we used an habituation-deshabituation test, where mice are habituated for three days to a cotton bud smelling of orange and challenged on the fourth day with a cotton bud smelling of vanilla which should raise their interest. Since mice are able to see the cotton bud at all time, their interest will only be raised if they can actually smell the difference. Indeed, normal mice became progressively used to the orange-smelling cotton bud and investigated it less every day, but on the fourth day their interest was sharply raised when a new odour was presented (Fig 4C, orange line). In contrast, and as predicted from the results of the buried food test, the interest of the PLD2 KO mice was not significantly increased by the vanilla-scented cotton bud, confirming that the mice had lost their sense of smell (Fig 4C blue line, p<0.001). Importantly, if the mice had been anosmic from birth, there would be increased mortality of new-born pups because they rely on their sense of smell to access milk [33]; this was not the case for the PLD2KO mice. Intriguingly, we found that the onset of anosmia is progressive and sets in between 11 and 14 weeks, as 11 week old PLD2KO mice were undistinguishable from WT mice in the habituation-deshabituation test (Fig 4D). As a control, we also tested the ability of mice to recognise two different sets of objects that share the same odour, to determine if PLD2KO mice are just uninterested in a new stimuli. We thus used a habituation-deshabituation test on glass marbles, a large clear one for the first three days and a pyramid of four small dark blue ones on the last day. We did not see any difference between WT and PLD2KO mice, indeed their interest was barely raised by the fourth day stimulus.

Anosmia can be triggered by a large number of factors–genetic [34] or environmental such as trauma- and can be caused by either a problem in the primary olfactory circuit, i.e. absence of input from the olfactory epithelium to the mitral cells, or their inability to respond to such input, or a more central problem [35]. To address these possibilities, we challenged 18 week old PLD2KO and WT mice with a variety of smells, quickly isolated their olfactory bulbs, sectioned them and stained with an antibody against c-fos, a marker for neuron activation. If the primary circuit had been malfunctioning, we would expect the PLD2 KO mice to have less c-fos-positive cells in the mitral cell layer than WT mice. On the other hand, if the problem is more central, there should be no difference between the two. Analysis of mice challenged with vanilla, chocolate or urine from the opposite sex did not show any difference in the distribution of c-fos-positive mitral cells (Fig 4B and 4B’), suggesting that the olfaction deficiency is related to a more central problem, possibly within the hippocampus as it is a target of the mitral cells through the entorhinal cortex.

## Discussion

The phospholipase D2 knock out mouse has previously been described as a model with increased resistance to Alzheimer’s insult [18]. In keeping with this paper, we provide evidence that PLD2 is expressed widely in the brain, though with varying degrees of expression in the neurons and an absence in glial-rich regions. We have investigated the brain of the PLD2KO mice and determined multiple phenotypes at the biochemical, histological and behavioural levels. Lipidomic analysis of isolated brain regions revealed that the quantitatively normal pool of PA is qualitatively and regionally abnormal, with a small but significant shift of PA pools from the shorter 32: and 34: PA families towards the longer 36: and 38: species. We take comfort in observing a similar trend in the lipidomic data previously presented by Oliveira et al [18]: namely a relative increase in 36: PA species and lowering of 34: species. This suggests that loss of PLD2 has an effect that is compensated overall in quantity by other PA-producing enzymes, but that the molecular species produced are not identical.

Considering that PLD2 is mostly located at the plasma membrane, it is likely that this cellular membrane will be most extensively affected by modifications in the abundance of particular PA species. This apparently small difference in composition has non-negligible effects on brain development and function. This is likely related to the consequent changes in membrane fluidity and curvature that can be induced by the differences in PA acyl structure. The lack of change in PIP_2_ species or content in those tissues in which the loss of PLD2 affected PA molecular species makeup implies that the change in the composition of PA did not affect its ability to interact with and thus regulate the activity of target proteins. It is therefore most likely that the change in PA species, will induce changes in both the bilayer thickness and in curvature and thereby modify cell function including cell motility [2]. Thus, we detected consistent ectopia of Purkinje cells in the white matter and on the surface of the molecular layer in the cerebellum, although this did not overtly affect the motor skills of the PLD2KO mice when tested using the rotarod paradigm. Finally, we found that age-related anosmia develops in PLD2KO mice, even though the primary circuit from the olfactory epithelium to the mitral cells in the olfactory bulb appeared normal, as judged by c-fos staining. This latter result suggests that it is not olfaction *per se* that is affected by the loss of PLD2, but its central processing. To our knowledge, the combination of Purkinje cells ectopia and olfaction problems have only previously been described as phenotypes in mice where the reelin pathway is perturbed [36–38]. It is notable that the receptors for this pathway are two lipoprotein receptors, VLDLR (Very Low Density Lipoprotein Receptor) and ApoER2 (Apolipoprotein E2 Receptor), the latter of which has been implicated in Alzheimer’s disease [39]. We have analysed the expression of reelin, VLDLR and ApoER2 by Western blotting, but have not detected any differences between PLD2KO and WT mice. However, it is possible that the observed phenotypes are independent of change in the levels of these protein, but rather how they function and how they transmit the signal. For example, PLD2 has been implicated in EGF and mu-opioid receptor endocytosis following ligand activation [4–6, 40]. It is therefore possible that PLD2 plays a role in the reelin pathway by facilitating signal transduction by endocytosis of the receptors, again pointing to selective effects of PA species change upon membrane properties. Alternatively, it is possible that a more systemic problem in dealing with lipids is indirectly causing the brain phenotypes. This would resonate with a recent report that rats and mice on high fat diet lose their sense of smell [41]. Further investigation is required to tease out these hypotheses.

Our results disagree in some respects with the PLD2 KO presented by Burkhardt et al [12]. Using MRI scans, they found that young and juvenile PLD2 KO brain development is significantly delayed which results in brain up to 50% smaller compared to WT controls. However, at P33, PLD2KO brains appear to have caught up and are no longer different volume wise from WT brains. We did not observe such a striking phenotype between WT and PLD2KO siblings in our mice neither was it reported by Oliveira et al. [18] in their knock out. This discrepancy however could be explained by strain difference. In conclusion, we complement the discovery by others that PLD2KO mice are resistant to Alzheimer’s insult [18] by showing that these mice also suffer from anosmia and subtle cytoarchitectural abnormalities that may phenocopy mice with deficiency in the reelin pathway. We speculate that the two are linked, probably through the receptors involved: VLDLR and ApoER2.

## Supporting Information

S1 File Contains full lipidomic data covering two separate experiments where the relative amount of PA species has been analysed in different brain regions. For each experiment, brain regions are described using the following code: Mouse gender (Male, M, or Female, F), Genotype, brain region: *CRB*, Cerebellum (green); *CTX*, Cortex (blue); *OLF*, Olfactory bulbs (purple); *TH*, Thalamus-Hypothalamus and Striatum (white); *HP*, Hippocampus; *Rest*, Midbrain-Hindbrain and Medulla (pink). Univariate Analysis of Variance is presented for each condition.
(XLSX)

## References

[1] Raghu P, Manifava M, Coadwell J, Ktistakis NT. Emerging findings from studies of phospholipase D in model organisms (and a short update on phosphatidic acid effectors). Biochimica et Biophysica Acta (BBA)—Molecular and Cell Biology of Lipids. 2009;1791(9):889–97. doi: 10.1016/j.bbalip.2009.03.013. 19345277

[2] Rudge SA, Wakelam MJO. Inter-regulatory dynamics of Phospholipase D and the Actin cytoskeleton. Biochim Biophys Acta. 2009;1791:856–61. 10.1016/j.bbalip.2009.04.008 19422932

[3] Selvy PE, Lavieri RR, Lindsley CW, Brown HA. Phospholipase D: Enzymology, Functionality, and Chemical Modulation. Chemical Reviews. 2011;111(10):6064–119. 10.1021/cr200296t 21936578 PMC3233269

[4] Du G, Huang P, Liang BT, Frohman MA. Phospholipase D2 Localizes to the Plasma Membrane and Regulates Angiotensin II Receptor Endocytosis. Molecular Biology of the Cell. 2004;15:1024–30. 14718562 10.1091/mbc.E03-09-0673 PMC363061

[5] Koch T, Brandenburg LO, Liang Y, Schulz S, Beyer A, Schroder H, et al Phospholipase D2 modulates agonist-induced mu-opioid receptor desensitization and resensitization. Journal of neurochemistry. 2004;88(3):680–8. Epub 2004/01/15. . 14720217 10.1046/j.1471-4159.2003.02189.x

[6] Zhao C, Du G, Skowronek K, Frohman MA, Bar-Sagi D. Phospholipase D2-generated phosphatidic acid couples EGFR stimulation to Ras activation by Sos. Nature Cell Biology. 2007;9(6):706–12. 17486115 10.1038/ncb1594

[7] Bader MF, Vitale N. Phospholipase D in calcium-regulated exocytosis: lessons from chromaffin cells. Biochim Biophys Acta. 2009;1791(9):936–41. Epub 2009/03/18. 10.1016/j.bbalip.2009.02.016 . 19289180

[8] Brown FD, Thompson NT, Saqib KM, Clark JM, Powner D, Thompson NT, et al Phospholipase D1 localises to secretory granules and lysosomes and is plasma-membrane translocated on cellular stimulation. Current Biology. 1998;8:835–8. 9663393 10.1016/s0960-9822(98)70326-4

[9] Vitale N. Synthesis of fusogenic lipids through activation of phospholipase D1 by GTPases and the kinase RSK2 is required for calcium-regulated exocytosis in neuroendocrine cells. Biochemical Society transactions. 2010;38(Pt 1):167–71. Epub 2010/01/16. 10.1042/bst0380167 . 20074053

[10] Colley WC, Sung T-C, Roll R, Jenco J, Hammond SM, Altshuller Y, et al Phospholipase D2, a distinct phospholipase D isoform with novel regulatory properties that provokes cytoskeletal reorganisation. Current Biology. 1997;7(3):191–201. 9395408 10.1016/s0960-9822(97)70090-3

[11] Pettitt TR, McDermott M, Saqib KM, Shimwell N, Wakelam MJO. Phospholipase D1b and D2a generate structurally identical phosphatidic acid species in mammalian cells. Biochem Journal. 2001;360:707–15. 11736663 10.1042/0264-6021:3600707 PMC1222276

[12] Burkhardt U, Stegner D, Hattingen E, Beyer S, Nieswandt B, Klein J. Impaired brain development and reduced cognitive function in phospholipase D-deficient mice. Neuroscience letters. 2014;572:48–52. Epub 2014/05/13. 10.1016/j.neulet.2014.04.052 . 24813107

[13] Burkhardt U, Wojcik B, Zimmermann M, Klein J. Phospholipase D is a target for inhibition of astroglial proliferation by ethanol. Neuropharmacology. 2014;79:1–9. Epub 2013/11/23. 10.1016/j.neuropharm.2013.11.002 . 24262632

[14] Chen Q, Hongu T, Sato T, Zhang Y, Ali W, Cavallo J-A, et al Key Roles for the Lipid Signaling Enzyme Phospholipase D1 in the Tumor Microenvironment During Tumor Angiogenesis and Metastasis. Science Signaling. 2012;5(249):1–9. 10.1126/scisignal.2003257 PMC3721670 23131846

[15] Dall'Armi C, Hurtado-Lorenzo A, Tian H, Morel E, Nezu A, Chan RB, et al The phospholipase D1 pathway modulates macroautophagy. Nature Communications. 2010;1(142). 10.1038/ncomms1144 PMC3328354 21266992

[16] Elvers M, Stegner D, Hagedorn I, Kleinschnitz C, Braun A, Kuijpers MEJ, et al Impaired a_llb_b_3_ Integrin Activation and Shear-Dependent Thrombus Formation in Mice Lacking Phospholipase D1. Science Signaling. 2010;3(103):1–10. 10.1126/scisignal.2000551 PMC3701458 20051593

[17] Norton LJ, Zhang Q, Saqib KM, Schrewe H, Macura K, Anderson KE, et al PLD1, rather than PLD2, regulates phorbol ester-, adhesion dependent-, and Fcg receptor-stimulated reactive oxygen species production in neutrophils. J Cell Science. 2011;124:1973–83. 10.1242/jcs.082008 21610093 PMC3104032

[18] Oliveira TG, Chan RB, Tian H, Laredo M, Shui G, Staniszewski A, et al Phospholipase D2 Ablation ameliorates Alzheimer's Disease-Linked Synaptic Dysfunction and Cognitive Deficits The Journal of Neuroscience. 2010;30:16419–28. 10.1523/JNEUROSCI.3317-10.2010 21147981 PMC3004537

[19] Silahtaroglu AN, Nolting D, Dyrskjot L, Berezikov E, Moller M, Tommerup N, et al Detection of microRNAs in frozen tissue sections by fluorescence in situ hybridization using locked nucleic acid probes and tyramide signal amplification. Nature Protocols. 2007;2(10):2520–8. Epub 2007/10/20. 10.1038/nprot.2007.313 . 17947994

[20] Tomomura M, Rice DS, Morgan JI, Yuzaki M. Purification of Purkinje cells by fluorescence-activated cell sorting from transgenic mice that express green fluorescent protein. The European journal of neuroscience. 2001;14(1):57–63. Epub 2001/08/08. . 11488949 10.1046/j.0953-816x.2001.01624.x

[21] Glynn D, Bortnick RA, Morton AJ. Complexin II is essential for normal neurological function in mice. Human molecular genetics. 2003;12(19):2431–48. Epub 2003/08/14. 10.1093/hmg/ddg249 . 12915444

[22] Clark J, Anderson KE, Juvin V, Smith TS, Karpe F, Wakelam MJO, et al Quantification of PtdInsP_3_ molecular species in cells and tissues by mass spectrometry. Nature Methods. 2011;8(3):267–72. 10.1038/nmeth.1564 21278744 PMC3460242

[23] Jarquin-Pardo M, Fitzpatrick A, Galiano FJ, First EA, Davis JN. Phosphatidic acid regulates the affinity of the murine phosphatidylinositol 4-phosphate 5-kinase-Ibeta for phosphatidylinositol-4-phosphate. Journal of Cellular Biochemistry. 2007;100(1):112–28. Epub 2006/08/05. 10.1002/jcb.21027 . 16888807

[24] Kim H, Lee J, Kim S, Shin MK, Min DS, Shin T. Differential expression of phospholipases D1 and D2 in mouse tissues. Cell Biology International. 2007;31:148–55. 17085061 10.1016/j.cellbi.2006.09.020

[25] Kim M, Moon C, Kim H, Shin MK, Min do S, Shin T. Developmental levels of phospholipase D isozymes in the brain of developing rats. Acta histochemica. 2010;112(1):81–91. Epub 2008/11/18. 10.1016/j.acthis.2008.09.004 . 19010519

[26] Kodaki T, Yamashita S. Cloning, Expression, and Characterization of a Novel Phospholipase D Complementary DNA from Rat Brain. Journal of Biological Chemistry. 1997;272(17):11408–13. 9111050 10.1074/jbc.272.17.11408

[27] Lopez I, Arnold RS, Lambeth JD. Cloning and Initial Characterization of a Human Phospholipase D2 (hPLD2). Journal of Biological Chemistry. 1998;273(21):12846–52. 9582313 10.1074/jbc.273.21.12846

[28] Lein ES, Hawrylycz MJ, Ao N, Ayres M, Bensinger A, Bernard A, et al Genome-wide atlas of gene expression in the adult mouse brain. Nature. 2007;445(7124):168–76. Epub 2006/12/08. 10.1038/nature05453 . 17151600

[29] Zhang Y, Huang P, Du G, Kanaho Y, Frohman MA, Tsirka SE. Increased expression of two phospholipase D isoforms during experimentally induced hippocampal mossy fiber outgrowth. Glia. 2004;46(1):74–83. Epub 2004/03/05. 10.1002/glia.10322 . 14999815

[30] Rogers DC, Fisher EM, Brown SD, Peters J, Hunter AJ, Martin JE. Behavioral and functional analysis of mouse phenotype: SHIRPA, a proposed protocol for comprehensive phenotype assessment. Mammalian genome: official journal of the International Mammalian Genome Society. 1997;8(10):711–3. Epub 1997/10/08. . 9321461 10.1007/s003359900551

[31] Hall CS. Emotional behaviour in the rat. I Defecation and urination as measures of individual differences in emotionality. Journal of Comparative Psychology. 1938;18:385–403.

[32] Jones BJ, Roberts DJ. The quantiative measurement of motor inco-ordination in naive mice using an acelerating rotarod. The Journal of pharmacy and pharmacology. 1968;20(4):302–4. Epub 1968/04/01. . 4384609 10.1111/j.2042-7158.1968.tb09743.x

[33] Brunet LJ, Gold GH, Ngai J. General anosmia caused by a targeted disruption of the mouse olfactory cyclic nucleotide-gated cation channel. Neuron. 1996;17(4):681–93. Epub 1996/10/01. . 8893025 10.1016/s0896-6273(00)80200-7

[34] Keydar I, Ben-Asher E, Feldmesser E, Nativ N, Oshimoto A, Restrepo D, et al General olfactory sensitivity database (GOSdb): candidate genes and their genomic variations. Human mutation. 2013;34(1):32–41. Epub 2012/09/01. 10.1002/humu.22212 22936402 PMC3627721

[35] Doty RL. Olfactory dysfunction in Parkinson disease. Nature reviews Neurology. 2012;8(6):329–39. Epub 2012/05/16. 10.1038/nrneurol.2012.80 . 22584158

[36] Krueger DD, Howell JL, Hebert BF, Olausson P, Taylor JR, Nairn AC. Assessment of cognitive function in the heterozygous reeler mouse. Psychopharmacology. 2006;189(1):95–104. Epub 2006/09/16. 10.1007/s00213-006-0530-0 16977475 PMC1618791

[37] Larouche M, Beffert U, Herz J, Hawkes R. The Reelin receptors Apoer2 and Vldlr coordinate the patterning of Purkinje cell topography in the developing mouse cerebellum. PloS one. 2008;3(2):e1653 Epub 2008/02/28. 10.1371/journal.pone.0001653 18301736 PMC2242849

[38] Larson J, Hoffman JS, Guidotti A, Costa E. Olfactory discrimination learning deficit in heterozygous reeler mice. Brain research. 2003;971(1):40–6. Epub 2003/04/15. . 12691835 10.1016/s0006-8993(03)02353-9

[39] Holtzman DM, Herz J, Bu G. Apolipoprotein E and apolipoprotein E receptors: normal biology and roles in Alzheimer disease. Cold Spring Harbor perspectives in medicine. 2012;2(3):a006312 Epub 2012/03/07. 10.1101/cshperspect.a006312 22393530 PMC3282491

[40] Shen Y, Xu L, Foster DA. Role for phospholipase D in receptor-mediated endocytosis. Molecular and Cellular Biology. 2001;21(2):595–602. Epub 2001/01/03. 10.1128/mcb.21.2.595-602.2001 11134345 PMC86627

[41] Thiebaud N, Johnson MC, Butler JL, Bell GA, Ferguson KL, Fadool AR, et al Hyperlipidemic diet causes loss of olfactory sensory neurons, reduces olfactory discrimination, and disrupts odor-reversal learning. The Journal of neuroscience: the official journal of the Society for Neuroscience. 2014;34(20):6970–84. Epub 2014/05/16. 10.1523/jneurosci.3366-13.2014 24828650 PMC4019806

